# Advancements in Glaucoma Diagnosis: The Role of AI in Medical Imaging

**DOI:** 10.3390/diagnostics14050530

**Published:** 2024-03-01

**Authors:** Clerimar Paulo Bragança, José Manuel Torres, Luciano Oliveira Macedo, Christophe Pinto de Almeida Soares

**Affiliations:** 1 ISUS Unit, Faculty of Science and Technology, University Fernando Pessoa, 4249-004 Porto, Portugal; jtorres@ufp.edu.pt (J.M.T.); csoares@ufp.edu.pt (C.P.d.A.S.); 2 Department of Ophthalmology, Eye Hospital of Southern Minas Gerais State, Rua Joaquim Rosa 14, Itanhandu 37464-000, MG, Brazil; lucianoomacedo@gmail.com; 3 Artificial Intelligence and Computer Science Laboratory, LIACC, University of Porto, 4100-000 Porto, Portugal

**Keywords:** deep learning, glaucoma, image analysis, artificial intelligence

## Abstract

The progress of artificial intelligence algorithms in digital image processing and automatic diagnosis studies of the eye disease glaucoma has been growing and presenting essential advances to guarantee better clinical care for the population. Given the context, this article describes the main types of glaucoma, traditional forms of diagnosis, and presents the global epidemiology of the disease. Furthermore, it explores how studies using artificial intelligence algorithms have been investigated as possible tools to aid in the early diagnosis of this pathology through population screening. Therefore, the related work section presents the main studies and methodologies used in the automatic classification of glaucoma from digital fundus images and artificial intelligence algorithms, as well as the main databases containing images labeled for glaucoma and publicly available for the training of machine learning algorithms.

## 1. Introduction

Glaucoma is a multifactorial neuropathy that can affect the fundus of the eye, causing gradual loss of vision and, in severe cases, blindness. Traditionally, the diagnosis of glaucoma is applied with the help of readily available ophthalmological teams and highly specialized equipment. The sensitivity of the diagnosis is generally high, as tests applied in ophthalmology offices have the clinical potential to identify virtually all cases of the disease. However, despite this sophisticated diagnostic scenario, the silent and slow evolution of the disease, the costs of exams and consultations, and the lack of access to public ophthalmological services in many cases prevent thousands of people from consulting an ophthalmologist during the early stages of this neuropathy. This contributes to the fact that around 70% of the patients are self-diagnosed, that is, alerted by their own visual impairment and not by an appropriate early diagnosis [1,2].

Glaucoma is considered a global problem; even in developed countries, it is estimated that at least 50% of patients with glaucoma do not know of their condition. This percentage is even worse in low-income countries [3]. It is considered a progressive, chronic, and incurable pathology; however, it can generally be efficiently controlled when treatment begins in the early stages of the disease.

There are several types of glaucoma: open-angle glaucoma, angle-closure glaucoma, congenital glaucoma and secondary glaucoma [4,5]. However, they all cause damage to the optic nerve, which in most cases occurs slowly, initially leading to the loss of midperipheral vision. In advanced stages, it affects central vision, leading to irreversible blindness.

**Figure 1:**
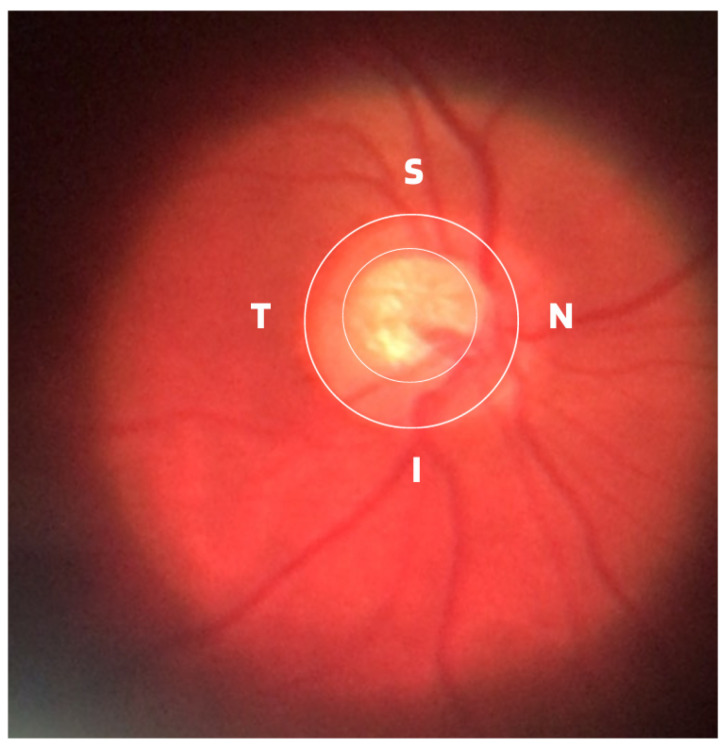
ISNT (Inferior (I), Superior (S), Nasal (N) and Temporal (T)) Rule.

Damage to the optic nerve can be analyzed using fundus examinations, also known as ophthalmoscopy or fundoscopy. The ophthalmoscopy examination is performed on the back part of the eye (fundus), which includes the retina, optic disc, choroid, and blood vessels. The funduscopic examination can be performed with a variety of equipment, such as direct ophthalmoscopy, indirect ophthalmoscopy, and slit lamp ophthalmoscopy. Found in almost all ophthalmology offices, these devices offer ophthalmologists a detailed view of the eyeball. As shown in Figure 1, the brightest part of the retina represents the optic disc (OD), which contains an excavation known as the optical cup (OC), depicted by the whitest part of the interior of the optic disc. Therefore, if the size of the optic cup increases, it is considered one of the main indicators of glaucoma [2,6,7,8].

In terms of the basic and traditional methods of diagnosing glaucoma, in addition to the fundus examination to examine the optic disc and the retinal nerve fiber layer (RNFL), ophthalmologists generally use tonometry and visual field tests as adjuncts. Tonometry is an exam to assess the degree of dysfunction and measures intraocular pressure (IOP) in millimeters of mercury (mmHg). The common eye pressure range is 10 to 21 mmHg, which is based on the average eye pressure level of a normal person. Although tonometry examination is very important in the management and treatment of glaucoma, it cannot be considered a diagnosis due to the presence of cases of normal pressure glaucoma [9]. Perimetry through the perimetry or campimetry exam, as is also known, the degree of functional impairment resulting from the disease is examined through the results of the obtained visual field map. In clinical practice, visual field testing identifies so-called blind spots (scotomas) and their locations in human vision and is therefore widely used as the gold standard to assess whether a patient suffers from typical functional glaucomatous damage [10].

**Figure 2:**
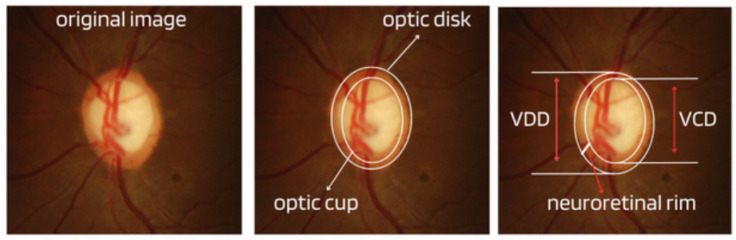
Measures considered in the CDR calculation.

**Figure 3:**
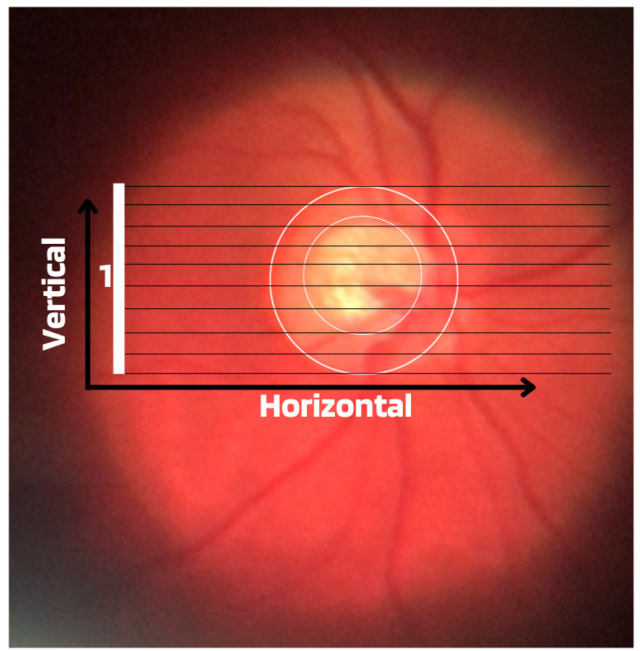
Example of CDR calculation with figure showing excavation of 0.6.

Although the demographic and clinical characteristics associated with glaucoma are relatively well known, there is still no uniform definition of the diagnosis of this disease by ophthalmologists. In this way, many international efforts have been made to develop such a definition, but no real consensus standard has been reached. Therefore, those with an IOP greater than 21 mmHg, accompanied by characteristic damage to the optic disc or defects in the visual field compatible with glaucoma, are generally included as glaucomatous [11]. Due to this particularity, it is important to assess and document the appearance of an increase in the cup-to-disc ratio as a way of evaluating possible structural damage caused by the disease, as well as accompanying the patient to treatment or routine appointments. Therefore, from ophthalmoscopy images, ophthalmologists can evaluate at least four important informative characteristics of glaucoma, such as cup/disc ratio, inferior (I), superior (S), nasal (N), and temporal (T) rule (ISNT), cup asymmetry, and in addition other structural damage caused to the optic disc, namely the following:

- Cup-to-Disc Ratio (CDR): An abnormal increase in disc cupping is important in the diagnosis of glaucoma; however, many people may have increased nerve cupping and not necessarily have glaucoma. This is especially true for myopic people, who tend to have a larger optical disc and consequently a larger optical cup. Therefore, during the diagnosis of glaucoma, it is important to assess not only the optical cup but also the cup-to-disc ratio (CDR). For better understanding, the CDR measurement is calculated from the relationship between the vertical diameter of the excavation (VCD) and the vertical diameter of the disc (VDD), as shown in Figure 2.
  To calculate the CDR ratio, the optical disc must be divided into 10 equal parts, as in Figure 3, and then the excavation scope must be taken into account in each division made. Therefore, it is considered a fractional percentage measurement, generally made horizontally, and can vary greatly between normal individuals. However, optical excavations greater than 0.65 indicate possible abnormalities, suggesting further investigation [2,12].
- ISNT Rule: The border formed between the optic cup and the optic disc, called the neuroretinal ring or neural ring, is also considered an indication of glaucoma, for which there is a rule called ISNT, which alludes to the orientation (inferior, superior, nasal, and temporal) of the edges in the image of the fundus, as shown in Figure 1. When considering the ISNT rule, in nonglaucomatous eyes, it is suggested that the thickness of the neural ring should be greatest in the inferior quadrant, followed by the superior, nasal, and temporal quadrants. Misalignment in the guidelines of this rule leads to suspicion of glaucoma [13].
- Cup-to-disc ratio (CDR) asymmetry: The CDR relationship between both eyes is symmetric in most people, and asymmetry is an important sign of suspected glaucomatous damage. This is due to the observation that 1% to 6% normal adults may have a discrepancy of 0.2 in the cup/disc ratio, while 1% of the general population may have an asymmetry of 0.3. Therefore, cup asymmetry is a finding on ophthalmological examination that requires additional tests to rule out the presence of glaucoma or other possible complications [14,15].
- Other structural damage to the optic disc: The main descriptions of these types of damage related to glaucoma are as follows [2,16,17]:
  1. Changes in RNFL: the presence of defects located in the retinal nerve fiber layer is called Hoyt’s sign and is characterized by a dark area that extends and widens from the optic disc, exhibiting an arched shape.
  2. Peripapillary atrophy: According to the ophthalmological appearance, peripapillary atrophy can be divided into a peripheral alpha zone and a central beta zone. The alpha zone is characterized by patchy hypopigmentation and thinning of the layers of the chorioretinal tissue. It is laterally adjacent to the retina and medially in contact with the beta area, with the sclera and large choroidal vessels visible. In normal eyes, the alpha and beta areas are usually located in the temporal area, followed by the inferior and superior areas. In glaucomatous eyes, the beta area is more present in the temporal region and its extension is associated with thinning of the RNFL.
  3. Excavation of the optic disc: In addition to disc excavation, the neuroretinal ring or neural rim must also be observed, as excavation is influenced by the size of the optic disc.
  4. Disc hemorrhage: The presence of peripapillary hemorrhages is an important sign in both the diagnosis and the monitoring of glaucoma. Therefore, vessel deflection and nasal excavation must be examined.
  5. Denudation of the lamina, cribriform: the presence of visible extinction of the cribriform lamina to the edge of the optic disc is called a notch, which represents the evolution of a defect located in the neural rim until there is a complete absence of tissue in the region, which exposes the cribriform lamina and allows visualization of its pores. Although it is very suggestive of glaucoma, this sign is not characteristic of the disease.

Regarding the difficulties associated with the diagnosis of glaucoma, it is considered that in cases in the moderate or advanced stages of the disease, the diagnosis is usually more simplified. However, the best way is to detect early glaucoma, which is essential for adequate treatment, mainly because quality of life can be altered even with slight loss of visual field [18]. However, the early identification of this disease, although important, can be challenging for several reasons, including glaucomatous characteristics that can be ambiguous in the optic disc region, RNFL, or visual field results at the beginning of the disease.

Over the years, more sensitive tests have been developed to more reliably identify early loss of visual function in patients with glaucoma, and more sophisticated imaging devices have been created to identify the first signs of disease-induced structural damage to aid in precocious diagnosis. Among these devices, optical coherence tomography (OCT), laser scanning polarimetry, and confocal laser scanning ophthalmoscopy stand out [19,20]. Although devices have demonstrated a good ability to assist ophthalmologists in the diagnosis of glaucoma, few studies have specifically examined the use of such technologies early in the disease, making the early diagnosis of glaucoma a difficult task for specialists, even with the aid of sophisticated equipment [19].

Given the difficulties in diagnosing glaucoma early, what ophthalmology clinics have done to try to overcome this difficulty is a combination of functional and structural exams. Although functional changes may be detected before structural changes, in many cases the first detectable manifestation of glaucoma is a structural abnormality change in the optic disc and RNFL, which therefore requires that the tests be combined to establish probability levels of the presence or absence of the disease [9,12,18].

## 2. Epidemiology

According to the World Health Organization (WHO), at least 2.2 billion people around the world suffer from some type of visual impairment. In almost half of the cases, this deficiency could have been avoided or has not yet been treated. When considering these data, it is inferred that today millions of people live with visual impairment or blindness that could have been avoided but unfortunately were not.

Although the exact number is unknown, it is estimated that 11.9 million people worldwide have moderate or severe visual impairment or blindness due to eye diseases such as glaucoma, trachoma (an inflammatory condition that affects the conjunctiva and cornea), and diabetic retinopathy, a chronic complication of diabetes mellitus [21,22,23].

Visual impairment and blindness can have a major impact on the daily lives of people affected by such disabilities, since vision is the dominant sense for humans at all stages of life. However, research estimates that by 2030, around 95.4 million people worldwide will have glaucoma.

Visual impairment, in addition to being detrimental to patient quality of life, also presents a huge global financial burden, as demonstrated by previous research that estimated the costs of lost productivity. These costs can be divided into direct costs and indirect costs. Direct costs include medications, surgeries, medical consultations, hospitalizations, and complementary examinations. Indirect medical costs include mainly the economic impacts caused by visual impairment on work productivity.

Although glaucoma generally progresses slowly and is underdiagnosed worldwide, it is the most common cause of irreversible blindness globally, yet it can be prevented. The disease is considered preventable because, if detected early, there are ways to control it, but global statistics show that due to underdiagnosis, the result is a large number of blind people. This problem can be even more serious in low-income or underdeveloped countries, such as Brazil, considered by the World Inequality Lab report in 2018 [24] as one of the countries with the highest social and income inequality in the world, marked by extreme levels for many consecutive years.

Although statistical numbers of underdiagnosis in the general population combined with the need for early diagnosis to prevent blindness may suggest that glaucoma is a good candidate for population screening, studies have shown that, at least in countries such as the United Kingdom and Finland, the detection of population-based glaucoma using traditional diagnostic methods is not feasible due to the high cost of implementation and maintenance and the relatively low prevalence of the disease in the general population, which is approximately 3.5% [25,26]. Similarly, the US Preventive Services Task Force [27], with the support of the American Academy of Family Physicians [28], does not recommend screening for glaucoma in the primary care setting, citing insufficient evidence to assess its implications, benefits, or harms.

## 3. Scientific and Technological Advances in Artificial Intelligence

In recent years, scientific and technological advances have opened up a wide range of clinical and research opportunities in the field of ophthalmological care, which can help combat glaucoma. In this way, artificial intelligence technologies have proven effective in areas of medicine such as radiology, pathology, dermatology, etc. All of these studies are in related areas that share parallels with ophthalmology because of their deep roots in diagnostic imaging.

The term artificial intelligence is a technology that covers several areas of knowledge and generally refers to the development of computational systems capable of performing tasks that mimic human intelligence. More recently, through machine learning and algorithms known as artificial neural networks (ANN) and deep neural networks (DNN) many advances have been possible [29,30].

The concept of machine learning encompasses a variety of methodologies, such as random forests [31], K-nearest neighbors (KNN) [32], support vector machines (SVM) [33], naive bayes [34], and artificial neural networks [29]. All of these technologies are aimed at pattern recognition, statistical regression, and data classification processes. Among machine learning algorithms, deep learning technology stands out, which has been at the forefront of the development and advances in computing and big data in recent years, mainly with the introduction and development of convolutional neural network (CNN) networks, proposed by researcher Yann LeCun [35] and especially used in the areas of pattern recognition and digital image classification.

The networks presented are algorithms that require a lot of data for training, but often there are not enough data, especially when considering clinical information. Therefore, a widely used technique that allows neural networks to be applied to small data sets is the process of transfer learning, considered the method of transferring knowledge acquired during training in a certain domain (a database) to be applied in another domain, that is, another similar problem. In view of this, algorithms that offer this technology are called pre-trained. One of the conveniences of using pre-trained networks is that they already have defined weights; that is, the weights are initialized with values obtained from already completed training.

Still in transfer learning, the ImageNet Large Scale Visual Recognition Challenge (ILSVRC) is an annual competition run by the ImageNet team since 2010, in which research teams evaluate the performance of computer vision and machine learning algorithms on various transfer learning tasks. visual recognition, such as object classification and localization [36]. ImageNet is a project aiming to provide large libraries of images for use in pre-training algorithms to be used in various other tasks and has been fundamental for advancing research in computer vision and deep learning. This database contains more than 14 million images, divided into more than 20,000 categories.

Due to data deficiency and other purposes, generative adversarial networks (GANs) also emerged, a machine learning architecture that consists of two networks that ’fight’ against each other (damage to the environment). The potential of GANs is enormous because they can learn to imitate any data distribution in the following way: First, a neural network called a generator generates new data instances, while another neural network called a discriminator evaluates their authenticity. In this way, the generator produces false images in the hope that the false images will even be considered real by the discriminator. With this exchange of information, the generator learns to generate plausible data, while the discriminator learns to distinguish false data from the generator. The discriminator penalizes the generator for producing concrete results, and with this, the generator improves more and more.

Training of GANs networks is carried out using real data instances as positive and fake data instances created by the generator as negative. After training, the classifier classifies the real and fake generator data and propagates the discriminator loss through the discriminator network to update the weights [37].

All these artificial intelligence technologies, regardless of the difficulty in finding large sets of public data or the algorithmic model used, show the great commitment of researchers to spread scientific growth seeking to find valid and effective solutions in the diagnosis of glaucoma. In this way, with respect to the application of artificial intelligence to ophthalmology, in addition to studies aimed at the automatic diagnosis of glaucoma, this technology also focuses on studies on the diagnosis of diseases such as cataracts, age-related macular degeneration, diabetic retinopathy, and others, showing that there is a set of ophthalmological diseases that can receive greater attention considering the use of deep learning.

Regarding the ophthalmological scenario of glaucoma, the use of artificial intelligence appears as an auxiliary tool in the diagnosis of the disease by detecting changes present in the OCT results, the results of the visual field exam, and mainly in the images of the fundus. This is because, despite the potential to apply automation to different types of ophthalmic images, fundus images (i.e., images obtained with conventional ophthalmic equipment) have gained prominence in many related works due to the availability, quality, and cost effectiveness of acquisition.

## 4. Related Works

To prepare this review, the manuscripts were selected based on the titles and summaries of the artificial intelligence methods used to classify glaucoma from digital fundus images, therefore presenting some of the relevant scientific works published in recent years. The search for articles was applied to the main data platforms (Scopus, Web of Science, Google Scholar, Scielo and Medline). Due to the scope of this study, the search was limited to algorithms developed to analyze digital fundus images, mainly with the aid of CNN algorithms. Before presenting methods using deep learning, we describe the main public databases containing fundus images used by many of the related works described as instances for training and testing the classifiers, which are mostly supervised.

### 4.1. Main Public Databases

Table 1 describes some publicly found databases for work focused on classifying glaucoma using deep learning and digital images of the fundus obtained by conventional retinography with cameras. The viewing angle of each database is also described, as it determines the amount of fundus area that will appear close to the optical disc. Furthermore, to fill in the data in the table, only images labeled glaucoma and nonglaucoma from each reported database were considered.

### 4.2. Approaches Using Deep Learning

Based on the analysis of the literature that constitutes the related studies, it was observed that artificial intelligence models used in studies of this disease based on digital fundus images are generally applied in two specific ways: calculating CDR or identifying glaucoma patterns in the optic disc region.

**Figure 4:**
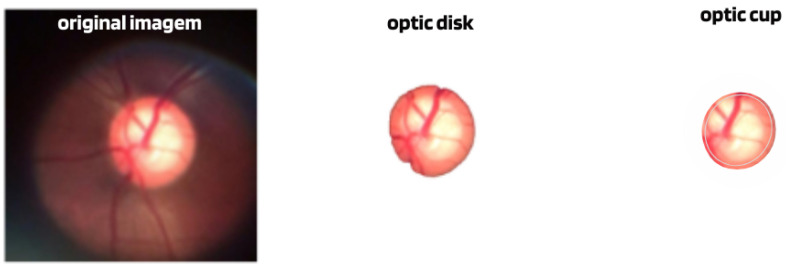
Example of image with segmentation of the disc and optical cup.

CDR calculation: One of the ways that glaucoma classification models have used has been through the calculation of the CDR measurement, generally obtained from the segmentation of the disc and optical cup structures; see Figure 4. The algorithms then, using the calculated CDR, estimate the presence or absence of glaucoma.

Although many algorithms, such as [52,53], have shown a high accuracy rate in segmenting these structures, this method can only be considered an indication of glaucoma and the need for a more detailed evaluation, since the diagnosis of this neuropathy is made by examination of the entire structure of the optic disc and not just excavation. Furthermore, although increased cupping suggests glaucoma, not all optic nerve cupping is related to this disease, as there are other conditions that can cause increased cupping of the optic nerve, such as neuritis, tumors, multiple sclerosis, etc.

Recognition of glaucomatous patterns: Although the CDR calculation algorithms only evaluate the excavation of the optic disc, this pattern recognition methodology seeks to evaluate the entire region of the optic disc in search of characteristics that could lead to the recognition of glaucoma. According to the context of deep learning and the analysis of related work described in this section, this type of application can be operated by at least four different methodologies, such as the following:

1. Feature vector extraction and classification: In this type of application, various image processing and feature extraction techniques can be used on digital images; however, a classifier will be the part of the system responsible for the categorization task, or that is, it will apply the decision process on which category a given image belongs to. Among the algorithms that work in this way are SVM, KNN, Naive Bayes, etc. Works such as these have been published by several authors and have appeared in [54,55].
2. Use of CNN networks: This approach eliminates the need to extract feature vectors, since CNN networks can extract such features through feature maps with their convolutional layers. Considered the gold standard of digital image processing, this methodology was applied in works such as those consulted in [38,56,57], using public and private databases.
3. Use of GANs networks: This involves discovering regularities and patterns in the input data and learning them automatically. Examples of these algorithms in glaucoma classification can be found in [58,59,60].
4. Use of multitechnologies: This type of modeling seeks to achieve the desired objective using a combination of techniques, such as KNN, SVM, CNN, etc. Numerous researchers, such as [61,62,63], have opted for this type of application, which is shown to be a valid way to recognize glaucomatous patterns.

Table 2 presents some of the various relevant works published in recent years as presented in reviews as available in Zedan et al. [64].

The benefits sought for these possible applications are varied, from the potential reduction in costs associated with the traditional diagnosis of glaucoma to assistance in population screening applications aimed at early diagnosis and reducing the rate of underdiagnosis of the disease.

## 5. Discussion and Conclusions

Significant progress has been made in the development of glaucoma classification algorithms, which have shown remarkable success in differentiating between digital fundoscopic images of glaucoma and nonglaucoma. According to the work of Phene et al. [76] in experiments using artificial intelligence in glaucoma classification, these algorithms have even shown higher precision compared to classifications made by experienced ophthalmologists. However, despite the consensus among various studies that artificial intelligence algorithms can be utilized as a supportive tool for the diagnosis of glaucoma, currently there is no software available for real clinical applications. This suggests that further theoretical and practical efforts are required to enhance the usability and effectiveness of such algorithms.

The machine learning methodology to achieve more representative tests faces challenges due to the limited number of images in the databases. In addition, the labeling process of these images can negatively affect the classification algorithms. In relation to database labeling, the studies discussed in this review generally required assessors (specifically ophthalmologists) to annotate labels by only examining retinal images to determine the presence or absence of glaucoma. However, a study involving six glaucoma specialists assigned to diagnose the disease solely on photographs of the ocular fundus revealed that their agreement was only 49% [77]. This finding highlights the fact that labeling the database solely based on fundus image observation can be detrimental to the classifier’s final results, as it is highly prone to errors. Consequently, training algorithms with inaccurately labeled data can compromise the overall quality of classifier results. To minimize errors in database insertion through image labeling, it is important to incorporate certain practices. One of such practice involves ensuring the presence of experienced ophthalmologists and adhering to the standard for the diagnosis of glaucoma, which entails a combination of functional and structural exams. Achieving this level of quality is often considered challenging. Consequently, some authors, such as Ting et al. [57] and Phene et al. [76] who work with large private datasets, have opted not to label their databases against a diagnostic gold standard. Instead, they have relied on a labeling consensus evaluated by experienced ophthalmologists. However, it should be noted that their databases were still labeled solely based on visual information obtained from fundus images.

The availability of images labeled with glaucoma in publicly accessible databases is limited in terms of quantity and diversity. These databases often consist of small sample sizes that are racially or clinically homogeneous, which may not accurately represent the entire population under study. Consequently, the applicability of algorithms to a broader context may be hindered. To address this limitation, researchers have explored the use of Generative Adversarial Networks (GANs) to generate synthetic images that resemble the original images. However, even if these networks produce satisfactory results, the generated images may not effectively address the issue of data homogeneity with respect to race or variations in the manifestation of glaucomatous damage. In light of these challenges, many authors opt to combine or merge multiple databases to improve the classification of glaucoma.

The exclusion of people with multiple eye injuries is an important consideration in the development of databases and glaucoma classification studies. Many authors have reported that they specifically removed individuals with ocular diseases other than glaucoma from their training and testing datasets. They also excluded images that were compromised by systemic diseases that could directly impact the optic nerve or visual field. However, this type of exclusion can be seen as a negative aspect, as it may manipulate the real-world scenario in favor of algorithmic precision. Furthermore, the racial homogeneity of the datasets contrasts with the diverse population, making it challenging to generalize the algorithms to populations beyond those observed in the dataset. However, when considering the quality of the databases and their construction, several key characteristics can be observed.

- The databases were obtained using high-resolution retinal cameras, except for the BrG set, which was obtained using a smartphone connected to a portable ophthalmoscope.
- With the exception of the refuge and Rim-one-dl datasets, which were formed using two digital fundus cameras, all other datasets were obtained using only one digital retinal camera.
- Most databases were labeled based on ophthalmological opinions solely by examining fundus images. Only a few databases were labeled with ophthalmic care and the gold standard for diagnosing glaucoma.
- All publicly available databases are considered too small to train classification algorithms from scratch, which means without using transfer learning.
- Publicly available databases generally have a homogeneous ethnic composition in the collected population.

In addition to the limitations of the database, deep learning algorithms face challenges in accurately classifying glaucoma due to the absence of consistent and objective diagnostic criteria. Consequently, researchers exploring the application of artificial intelligence in this field have had to establish their own definitions for categorizing instances as “yes” or “no” for glaucoma. As a result, various approaches have been pursued, such as texture analysis, analysis of the CDR ratio, ISNT rules, and others. This divergence in methods is mainly attributed to the absence of specific and quantifiable biomarkers to define the disease. Consequently, many researchers have attempted to predict similar diagnostic results for glaucoma, but have employed different methodologies, making it challenging to compare the performance of different studies. These biomarkers are essential not only to provide a definitive diagnosis, but also to justify the reasoning behind the diagnosis.

In light of this medical necessity, numerous authors, such as Ting et al. [57], have demonstrated the importance of identifying crucial image regions in order to validate the results obtained by deep learning algorithms when used for the classification of glaucoma. This approach serves as a justification for the results achieved by the methodology, at least until the healthcare community fully accepts these algorithms.

In the given context, it is important to note that the main objective of the previous studies was not to develop a market-ready algorithm, but rather to showcase the essential components required to achieve satisfactory results in glaucoma classification using fundus images. These findings may be valuable for potential future applications. As a result, for further advancement of such research, it is recommended to label databases based on the diagnostic gold standard in order to enhance the utilization of deep learning algorithms. In addition, there should be a clear distinction between training and test sets, with a diverse range of images captured by different devices, involving patients from various ethnic backgrounds. Furthermore, the databases should include images captured under different lighting conditions, contrast levels, noise levels, etc. [51,78].

After analyzing the databases identified, it can be observed that they only partially fulfill the requirements outlined in this study. However, they still play an important role in the training of various algorithms and driving technological advancement. In terms of the algorithms themselves, although some scientific research has demonstrated their high accuracy in distinguishing between glaucomatous and nonglaucomatous images, further clinical trials and in-depth studies are needed to identify and address potential factors that may hinder the integration of such algorithms into practical clinical applications. With continued efforts in this area, it is anticipated that future advances in artificial intelligence will greatly contribute to the diagnosis of eye diseases, including glaucoma.

## Figures and Tables

**Table 1:** Public and labeled databases for glaucoma.

Database	Glaucoma	Normal	Total	Viewing Angle
Acrima [38]	396	396	700	30 a 50°
Drions [39]	55	55	110	30 a 50°
Drishti-Gs1 [40]	50	51	101	30°
Drive [41]	34	6	40	45°
Glaucoma DB [42]	85	35	120	30 a 50°
Hrf [43]	15	15	30	45°
sjchoi86-Frf [44]	101	300	401	30 a 50°
Messidor [45]	28	72	100	45°
Origa [46]	168	482	650	30 a 50°
Papila [47]	155	333	488	30 a 50°
Refuge [48]	120	1080	1200	30 a 50°
G1020 [49]	296	724	1020	45°
BrG [50]	1000	1000	2000	25°
Rim-one DL [51]	172	313	485	30 a 50°

**Table 2:** Examples of work related to glaucoma classification using artificial intelligence algorithms.

Paper	Algorithm	Dataset	Accuracy/Precision
Dias et al. [38]	multilevel CNN	Private	99.4%
Bragança et al. [50]	Ensemble CNN	BrG	90.0%
Singh et al. [54]	SVM, KNN e Naive Bayes	STARE e MESSIDOR	95.0%
Shiny et al. [55]	SVM	DRISHTI	95.3%
Shinde et al. [61]	Le-Net e modelo U-Net CNN	RIM-ONE, DRISHTI-GS, DRIONS-DB, JSIEC e DRIVE	100%
Sreng et al. [62]	VGG16-19,Xception, ResNet50 e InceptionV3	ACRIMA, DRISHTI GS1, HRF, RIM-ONE,	96.5%
Santos et al. [63]	DeepLabv3+ and MobileNet	RIM-ONE, ORIGA, ACRIMA, DRISHTI-GS1 and REFUGE	95.59
Zulfira et al. [65]	SVM, KNN e Naive Bayes	DRIONS-DB	98.6%
Yunitasari et al. [66]	Dynamic Ensemble	RIM-ONE	91.0%
Wang et al. [67]	SVM	DRISHTI	95.0%
Gheisari et al. [68]	VGG e AlexNet	DRIONS-DB, HRF, RIM-ONE e DRISHTI-GS1	94.3%
Li et al. [69]	VGG, ResNet e RNN	Private	95.0%
Liu et al. [70]	ResNet	Private	95.0%
Nawaz et al. [71]	ResNet	Private	96.2%
Kim et al. [72]	EficienteNet-B0	ORIGA	97.2%
Hemelings et al. [73]	VGG, Inception e ResNet	Private	96.2%
Alghamdi et al. [74]	ResNet	Private	98.0%
Aamir et al. [75]	VGG-16	RIM-ONE e RIGA	93.0%

## Data Availability

This study explores the possibility of using artificial intelligence algorithms to help reduce the underdiagnosis of glaucoma. Therefore, a more general analysis of the state of the art is carried out, taking into account the subject discussed.
